# Flexible Memristor Devices Using Hybrid Polymer/Electrodeposited GeSbTe Nanoscale Thin Films

**DOI:** 10.1021/acsanm.2c03639

**Published:** 2022-11-25

**Authors:** Ayoub H. Jaafar, Lingcong Meng, Tongjun Zhang, Dongkai Guo, Daniel Newbrook, Wenjian Zhang, Gillian Reid, C. H. de Groot, Philip N. Bartlett, Ruomeng Huang

**Affiliations:** † School of Electronics and Computer Science, University of Southampton, Southampton, SO17 1BJ, United Kingdom; ‡ School of Physics and Astronomy, University of Nottingham, Nottingham, NG7 2RD, United Kingdom; § School of Chemistry, University of Southampton, Southampton, SO17 1BJ, United Kingdom; ∥ School of Chemistry, University of Lincoln, Lincoln, LN6 7TS, United Kingdom

**Keywords:** Flexible, electrodeposition, hybrid material, resistive switching, multilevel states

## Abstract

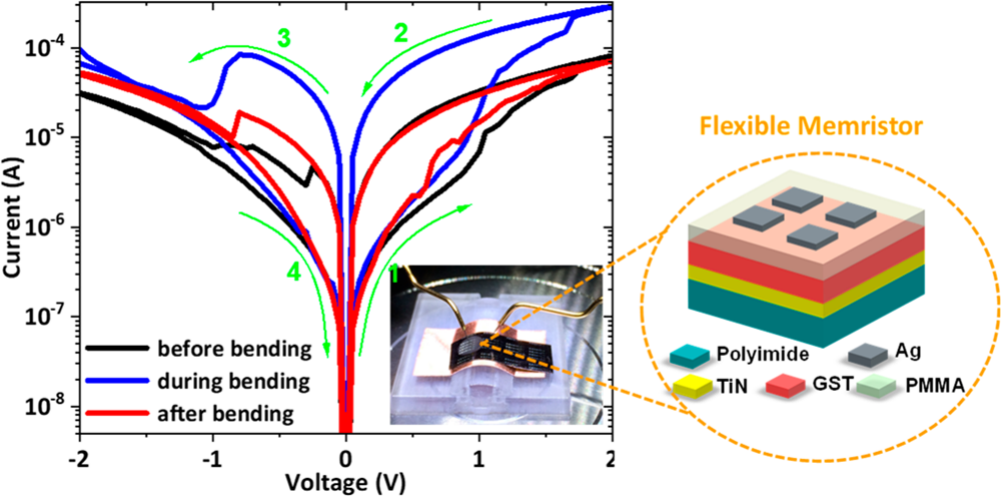

We report on the development of hybrid organic–inorganic material-based flexible memristor devices made by a fast and simple electrochemical fabrication method. The devices consist of a bilayer of poly(methyl methacrylate) (PMMA) and Te-rich GeSbTe chalcogenide nanoscale thin films sandwiched between Ag top and TiN bottom electrodes on both Si and flexible polyimide substrates. These hybrid memristors require no electroforming process and exhibit reliable and reproducible bipolar resistive switching at low switching voltages under both flat and bending conditions. Multistate switching behavior can also be achieved by controlling the compliance current (CC). We attribute the switching between the high resistance state (HRS) and low resistance state (LRS) in the devices to the formation and rupture of conductive Ag filaments within the hybrid PMMA/GeSbTe matrix. This work provides a promising route to fabricate flexible memory devices through an electrodeposition process for application in flexible electronics.

## 1 Introduction

Fabrication of modern electronic devices on a bendable substrate is essential for the future development of foldable, stretchable, and wearable electronic applications.^1^ This new class of electronics enables new product paradigms that are not possible with conventional semiconductors and rigid substrates, while offering the advantages of low cost and light weight.^2^ The past decades have witnessed remarkable progress for flexible electronics in many sectors including consumer electronics, automotive, retail, and healthcare.^3,4^ As a fundamental component of all modern electronic system, research on flexible electronic memory has attracted tremendous attention with several categories of memory devices proposed.^5^ Resistive switching based memristor devices can store and process information and are considered among the most promising candidates for next generation memory due to their simple structure, low power consumption, excellent storage capability, and scalability.^6−13^

Typically, a resistive memristor consists of a simple metal–insulator–metal (MIM) structure where the resistive switching material is sandwiched between two electrodes. The switching between the low resistance state (LRS) and high resistance state (HRS) is achieved by the creation and disruption of conductive filaments which are made of either metallic atoms from the active electrode or the defects within the switching layer.^14^ Memristors based on flexible substrates have been extensively researched over the past decade, while various materials have been investigated as the switching layer, such as organic materials,^15,16^ 2D materials,^17,18^ metal oxides,^19−21^ graphene oxide,^22^ and perovskites.^23^ Semiconductor chalcogenide based memristors have demonstrated increasing potential due to their excellent scalability and compatibility with CMOS circuits.^24^ The most widely used semiconductor chalcogenide GeSbTe has seen extensive usage in phase-change memory (PCM).^25^ The data storage in PCM relies on the rapid and reversible switch between the amorphous (high resistance) and crystalline (low resistance) phase of the material. This involves a melt-quenching reset process that requires a high programming current, which has become the major obstacle for the integration of PCMs.^26^ Resistive switching, however, does not involve major structural phase changes, and the switching between the HRS and LRS and vice versa can be achieved at lower voltages upon change the polarity of the applied electric field.^27^ Resistive switching effects have been observed in several chalcogenides such as AgGeSe, AgGeTe, AgInSbTe, and GeSbTe, with switching mechanisms attributed to a formation and rapture of conductive filaments either from the migration of metal cations (e.g., Ag and Cu)^28−36^ in the electrochemical metallization (ECM) process or from migration of Sb and Te ions inside the chalcogenide materials.^27,37−41^

Conventionally, chalcogenide materials are deposited via techniques such as physical vapor deposition, chemical vapor deposition, and atomic layer deposition.^42^ These “top-down” approaches often require vacuum equipment, which leads to high deposition costs and slow deposition rates. Additionally, their high thermal budgets can also be prohibitive for their application in flexible application. Electrodeposition is a well-established deposition method in the electronic industry that offers a low-cost and fast alternative solution for chalcogenide deposition.^43,44^ Our group has previously reported the electrodeposition of amorphous ternary GeSbTe thin films from a single, highly tunable, nonaqueous electrolyte,^45,46^ demonstrating both phase-change and resistive switching properties in highly scalable cross-bar architectures.^41,47^ More importantly, electrodeposition is a low-temperature deposition technique which makes it highly suitable for deposition onto flexible substrates. However, the deposition of a nanoscale thin film chalcogenide-based memristor onto flexible substrate via electrodeposition has never been reported.

In addition, hybrid organic–inorganic material-based memristors have attracted attention since they combine the electronic properties of inorganic components and the solution processing advantages of organic components.^48−53^ In comparison to inorganic material-based memristors, hybrid memristors have demonstrated improved switching properties such as high ON/OFF ratio, ultralow operating voltage, reduced power consumption, as well as having various advantages such as multilevel data storage, analogue switching, and flexibility.^54−59^

In this work, we report the development of high-quality hybrid memristor devices that are fabricated by electrodeposition. The hybrid memristor devices consist of a bilayer of electrodeposited GeSbTe nanoscale thin films combined with different thicknesses of an insulating PMMA layer (the thickness is controlled by changing the PMMA concentration) sandwiched between a TiN bottom electrode and an Ag top electrode using Si and polyimide substrates. The hybrid devices demonstrate desirable memristor properties such as a free forming process, low operation voltages, good cycling endurance, multistate switching behavior, and, importantly, mechanical flexibility. These properties open the route to realize fully the potential of memristor devices for low-cost and high-performance flexible electronics.

## 2 Results and Discussion

**Figure 1:**
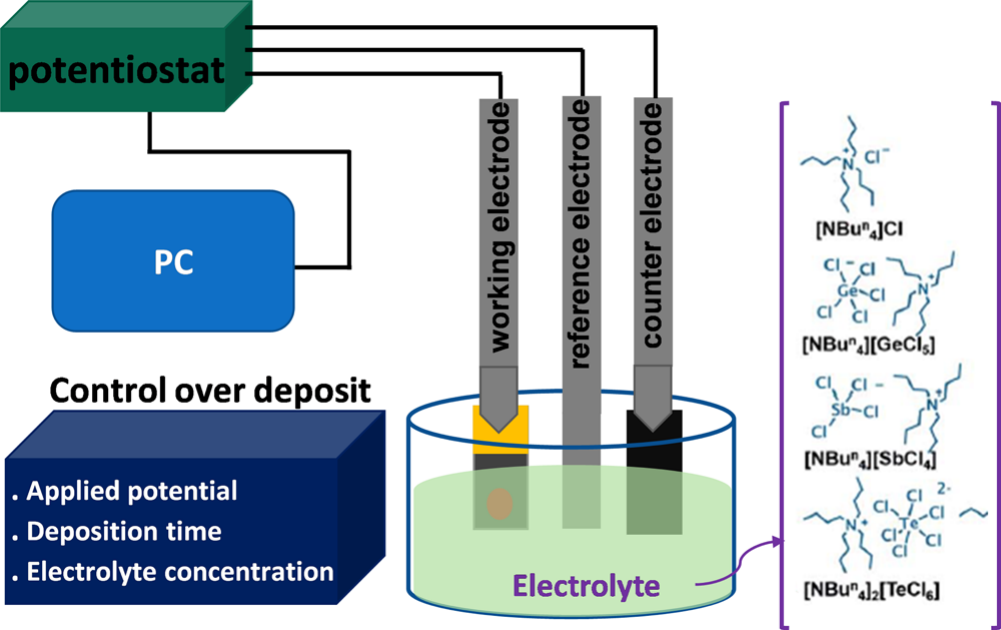
Schematic of the electrodeposition setup containing the chip connected through its global electrode to the potentiostat, together with the molecular structure of the precursors used in the dichloromethane solvent.

**Figure 2:**
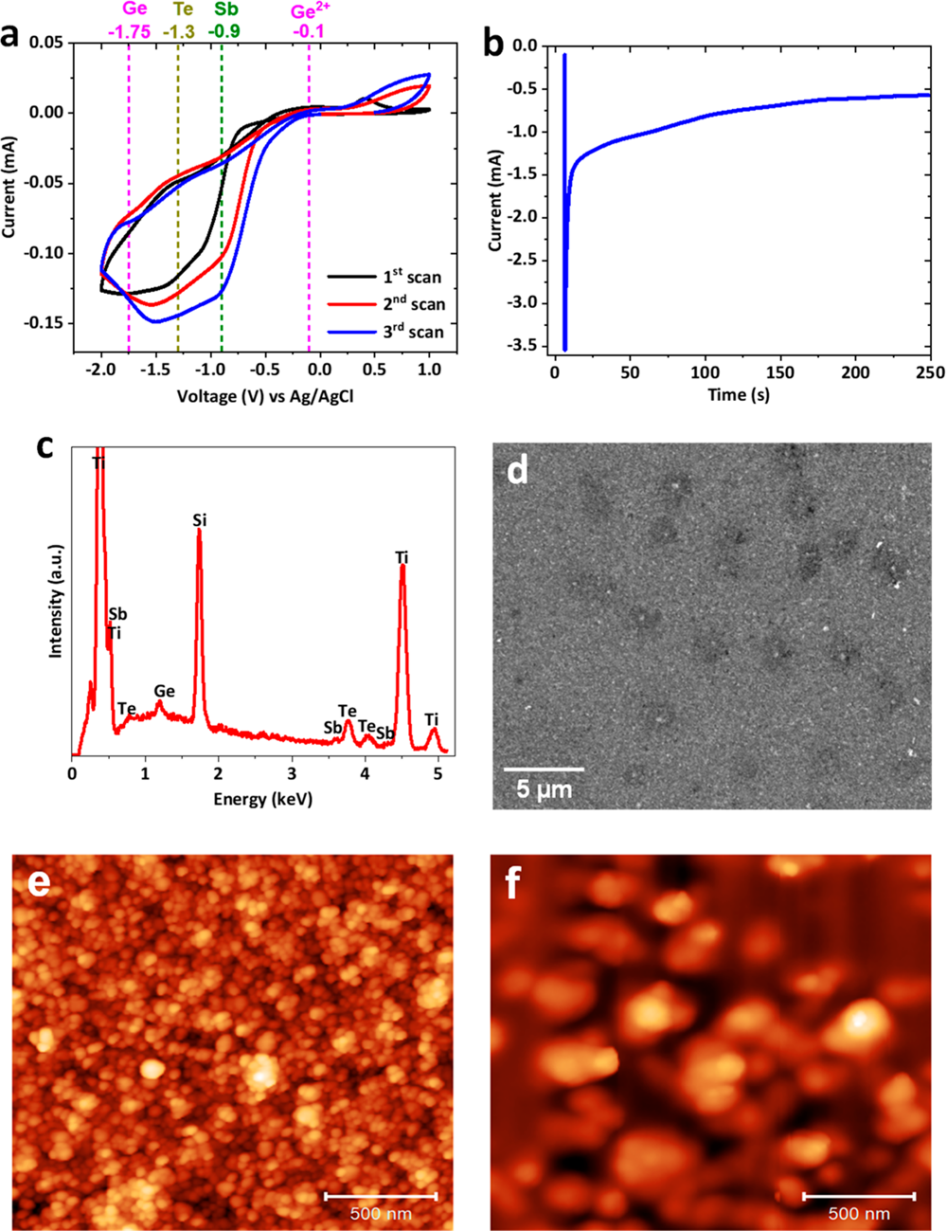
(a) Cyclic voltammogram of the 4-mm-diameter TiN/SiO_2_/Si substrate in 0.1 M [N^n^Bu_4_]Cl electrolyte containing 2.5 mM [N^n^Bu_4_]GeCl_5_, 1 mM [N^n^Bu_4_]SbCl_4_, and 2 mM [N^n^Bu_4_]_2_TeCl_6_. Scan rate: 50 mV s^–1^. (b) Current time transient for GeSbTe electrodeposition at a deposition potential of −1.75 V vs Ag/AgCl. The cutoff charge is −5 μC. (c) EDX spectrum of the electrodeposited GeSbTe on a Si substrate showing the presence of Ge, Sb, and Te within the thin film. (d) Top SEM image for GeSbTe thin film. (e) AFM image of GeSbTe thin film. (f) AFM image of PMMA(50%)/GeSbTe bilayer.

Figure 1 shows a schematic of the electrodeposition setup using a potentiostat connected to a TiN electrode through a Cr/Au global contact. The depositions were performed in a three-electrode cell including a Pt/Ir (90:10%) disc as a counter electrode and a Ag/AgCl wire in a glass frit containing 0.1 M [N^n^Bu_4_]Cl in dichloromethane (CH_2_Cl_2_) as a reference electrode. Electrodeposition of GeSbTe thin film was carried out in a dichloromethane (DCM) solution using precursors for Ge, Sb, and Te synthesized in-house.^60^ Prepatterned TiN thin films on Si/SiO_2_ substrates were used as the deposition electrode. The details of TiN electrode fabrication were reported in our previous work.^46^ Prior to the film deposition, cyclic voltammetry (CV) was recorded as shown in Figure 2a. The four shoulders at approximately −0.1 V, −0.9 V, −1.3 V, and −1.75 V correspond with the reduction of Ge^4+^ to Ge^2+^, Sb^3+^, Te^4+^, and Ge^2+^ to Ge(0), respectively.^47,61^ To ensure the incorporation of all three elements, the deposition potential of −1.75 V vs Ag/AgCl was selected. The detailed process of the electrodeposition is provided in the Experimental Section. The current–time transient of the deposition is shown in Figure 2b. Initial current spike is caused by double layer charging, followed by the current magnitude decreasing and reaching a steady state, indicating a diffusion controlled process. The film thickness was controlled by a cutoff charge of 5 μC to reach a nominal thickness of 100 nm.

The composition of the electrodeposited GeSbTe film was characterized by energy dispersive X-ray analysis (EDX) and the spectrum is presented in Figure 2c. The plot clearly shows the presence of all three elements in the GeSbTe thin film, without any significant impurities. The film is Te-rich, with a Ge:Sb:Te composition ratio of 12:10:78. Top view scanning electron microscopy (SEM) and atomic force microscopy AFM images of the electrodeposited GeSbTe thin film are shown in Figure 2d and e, respectively. The images show that the film is uniformly deposited on the substrate and consists of granules with sizes of ca. 50 nm. Poly(methyl methacrylate) (PMMA) films were subsequently coated on the GeSbTe films to form a PMMA/GeSbTe hybrid material (details in the Experimental Section). An AFM image of the hybrid material with 50% concentration of PMMA is shown in Figure 2f. Note that the roughness of the GeSbTe thin film, Figure 2e, is 9.38 nm, whereas the roughness of the PMMA/GeSbTe bilayer, Figure 2f, is 14.84 nm. The thickness of the PMMA film is controlled by the concentration of the PMMA solution (PMMA to anisole ratio), where a higher concentration will lead to a thicker film. The thickness of PMMA as a function of solution concentration is shown in the Supporting Information Figure S1. The memristor fabrication process is completed by patterning Ag top electrodes on the hybrid organic–inorganic structure using thermal evaporation technique.

**Figure 3:**
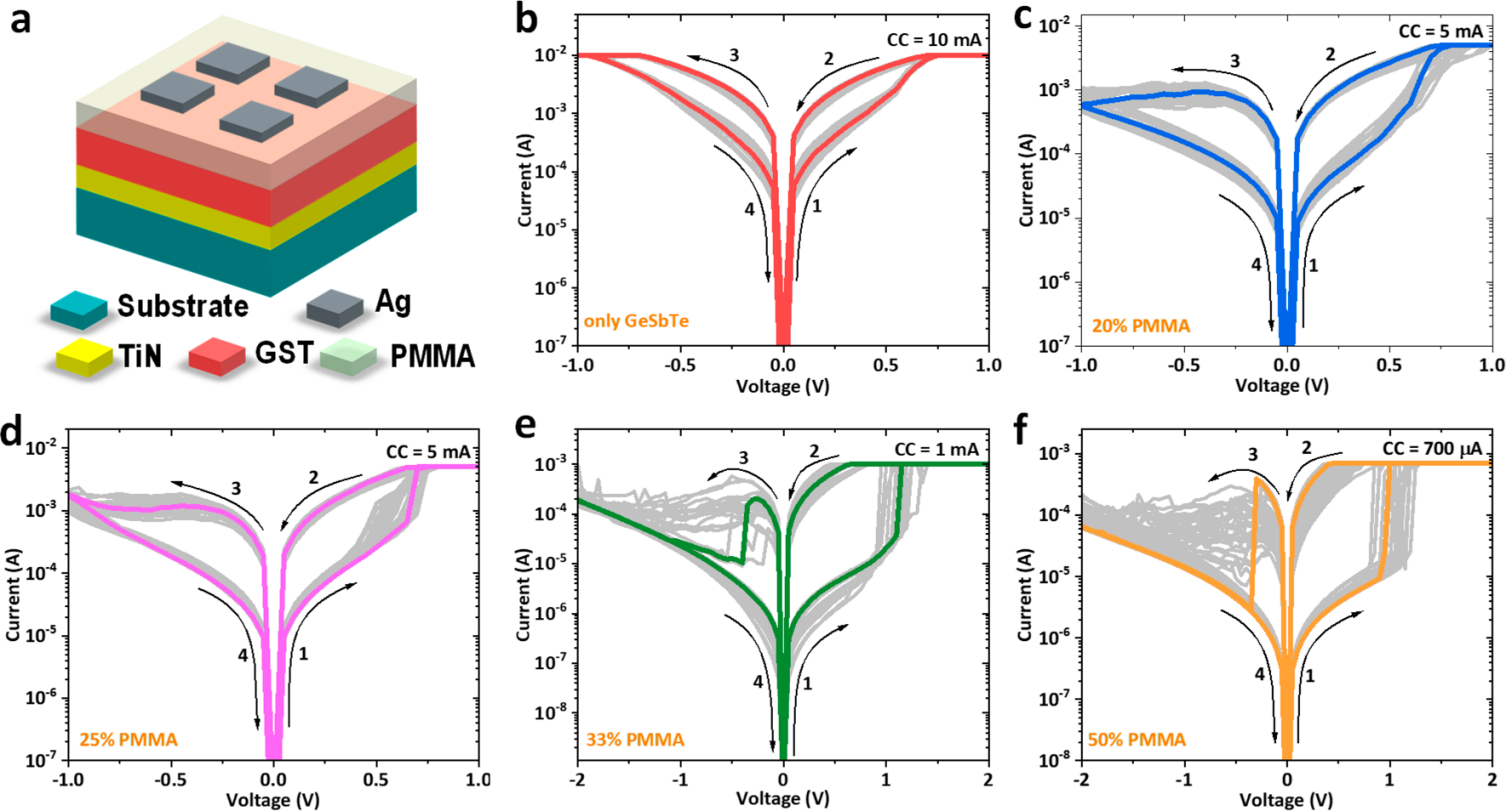
(a) Schematic of hybrid memristor devices. (b) *I*–*V* characteristics of a memristor consisting of a GeSbTe thin film (without PMMA) sandwiched between TiN and Ag electrodes, Ag/GeSbTe/TiN. (c–f) *I*–*V* characteristics of memristor consisting of hybrid PMMA/GeSbTe bilayer, Ag/PMMA/GeSbTe/TiN, with different concentrations of PMMA. The PMMA concentrations are 20% (c), 25% (d), 33% (e), and 50% (f).

Figure 3a schematically describes the developed hybrid organic–inorganic memristor based on the PMMA/GeSbTe bilayer structure. We first investigate the electronic switching properties of a single layer GeSbTe-based memristor without PMMA. The DC-IV characteristic of the device is shown in Figure 3b with the direction of the current sweep indicated by arrows. The device is initially in a high resistance state (HRS) and could be switched to a low resistance state (LRS) using a positive DC sweep from 0 to 1 V under a compliance current (CC) of 10 mA. The RESET from the LRS to HRS was achieved by a negative DC sweep from 0 to −1 V, representing a typical bipolar switching behavior. It is worth noting that a CC was needed at the RESET process to limit the operating current and to protect the device from breakdown.^41,62,63^ The absence of the CC during the RESET process switches the single-layer GeSbTe-based device to a higher operating current, resulting in breakdown of the device.^41^ Subsequent DC-IV cycles (gray lines) are similar to the initial switching cycle (red line). This indicates that no electroforming process is required to initialize the devices for the resistive switching effect. Note that the electroforming process is needed to initiate the switching in oxides.^64^ However, we expect the lack of the forming process in our devices due to a large amount of Te atoms within the Te-rich GeSbTe matrix, which can play a role in the formation of Te conductive filament(s). Both SET and RESET processes are characterized by gradual current change, and the device only operates at high current. Such high operating current is likely due to the formation of strong filament(s) within the GeSbTe layer. This is not surprising, as the granule-like texture of the as-deposited GeSbTe layer implies a certain amount of porosity in the film.^32,41^ It has been suggested that the voids or pores in the electrolyte layer could serve to facilitate ion migration in the thin film for the electrochemical metallization (ECM) memories.^65^ The voids or pores within the GeSbTe electrolyte can likely act as diffusion pathways for Ag cations, enabling the formation of conductive nano Ag filament(s) across the device.^32^ This high operating current leads to high power consumption which is highly undesirable for memristors.

The hybrid PMMA/GeSbTe-based memristor exhibits similar forming-free, bipolar resistive switching behavior to the single layer device (shown in Figure 3c–f). However, significant differences in the resistive switching properties can be observed. The most noticeable change is the lower CC required for the SET process. As the PMMA concentration in the solution increases from 20% to 50%, the CC required dropped by almost 1 order of magnitude from 5 mA to 700 μA with an associated reduction in power consumption. In addition, the voltage switching between the two resistance states changes from smooth to sharp for higher concentration of the PMMA layer. Another interesting feature is that all hybrid PMMA/GeSbTe-based devices do not require CC during the RESET process, facilitating subsequent circuit design. The DC endurance properties of the single layer and hybrid PMMA/GeSbTe devices are presented in Figure S2a–e. Retention testing for the hybrid TiN/PMMA/GeSbTe/Ag memristor (50% PMMA concentration) in both the HRS and LRS were examined at a continuous read voltage of 0.1 V for over 4000 s and are presented in Figure S2f.

**Figure 4:**
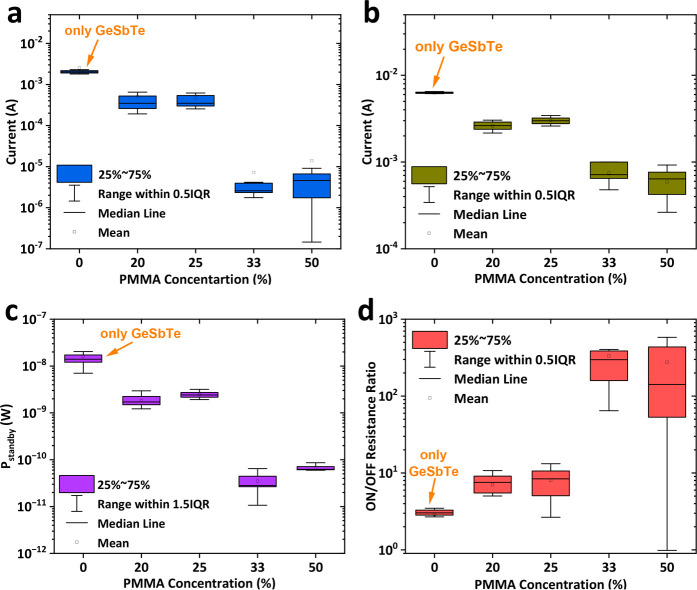
Resistive switching characteristics of hybrid Ag/PMMA/GeSbTe/TiN memristor devices on Si substrate. (a) OFF current versus PMMA concentration; (b) ON current versus PMMA concentration; (c) power consumption versus PMMA concentration; (d) ON/OFF ratio versus PMMA concentration. The OFF and ON currents and the ON/OFF resistance ratio were taken at a read voltage of 0.1 V.

To better understand the impact of the additional PMMA layer on the memristor switching properties, we plot the operating currents (at 0.1 V), the power consumption, and the ON/OFF resistance ratio of each device as a function of the PMMA concentration. Figure 4a,b shows the distribution of OFF and ON currents, respectively. The plots clearly show that increasing the PMMA concentration from 20% to 50% decreases both the OFF and ON currents. The plots also show the GeSbTe devices without the PMMA to have the highest OFF and ON currents, making our hybrid devices a promising candidate for low power consumption memories. Figure 4c shows that the power consumption reduces from 570 nW for the GeSbTe thin film to only 11 nW for the hybrid PMMA/GeSbTe device (50% PMMA). The power consumption, (*P*_Standby_ = *I*_HRS_ × *V*_read_), was calculated at a read voltage of (100 mV) and the OFF currents of the devices. This notable control of the currents results in a significant improvement in the separation between the OFF and ON states and therefore enhances the ON/OFF resistance ratio from 8 to 836, as shown in Figure 4d. All these comparisons clearly suggest that the switching performance of the inorganic GeSbTe memristor can be significantly improved by the incorporating of an organic layer.

**Figure 5:**
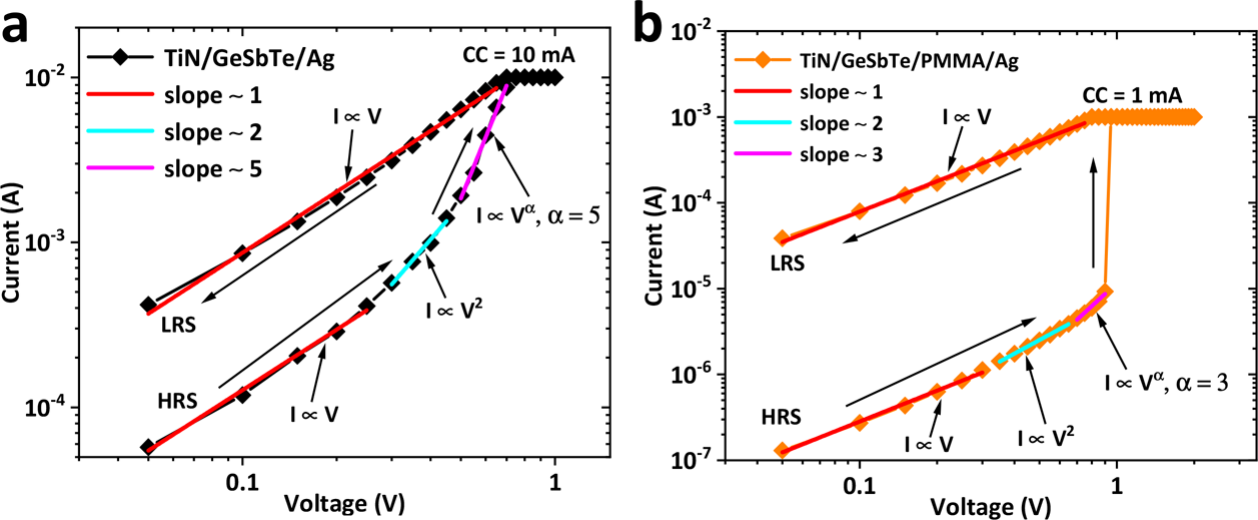
Current–voltage curves demonstrating fits to the SCLC mechanism for the HRS to LRS transition for a Ag/GeSbTe/TiN memristor device (a) and a hybrid Ag/PMMA/GeSbTe/TiN memristor device (50% PMMA) (b), both fabricated on Si substrates.

To explore the conduction mechanism in our control Ag/GeSbTe/TiN memristor and hybrid Ag/PMMA/GeSbTe/TiN memristor, the current–voltage curves were replotted in double-logarithmic scale as shown in Figure 5. The fitting results indicate a similar conduction mechanism for both devices. While the Ohmic conduction mechanism is dominant in the LRS state, the HRS state combines Ohmic conduction in the low applied voltage region, indicating conduction induced by thermally generated carriers, and space charge limited current (SCLC) in the high applied voltage region. A similar conduction mechanism is observed for the hybrid material-based devices with different concentrations of PMMA and is reported in Figure S3.

**Figure 6:**
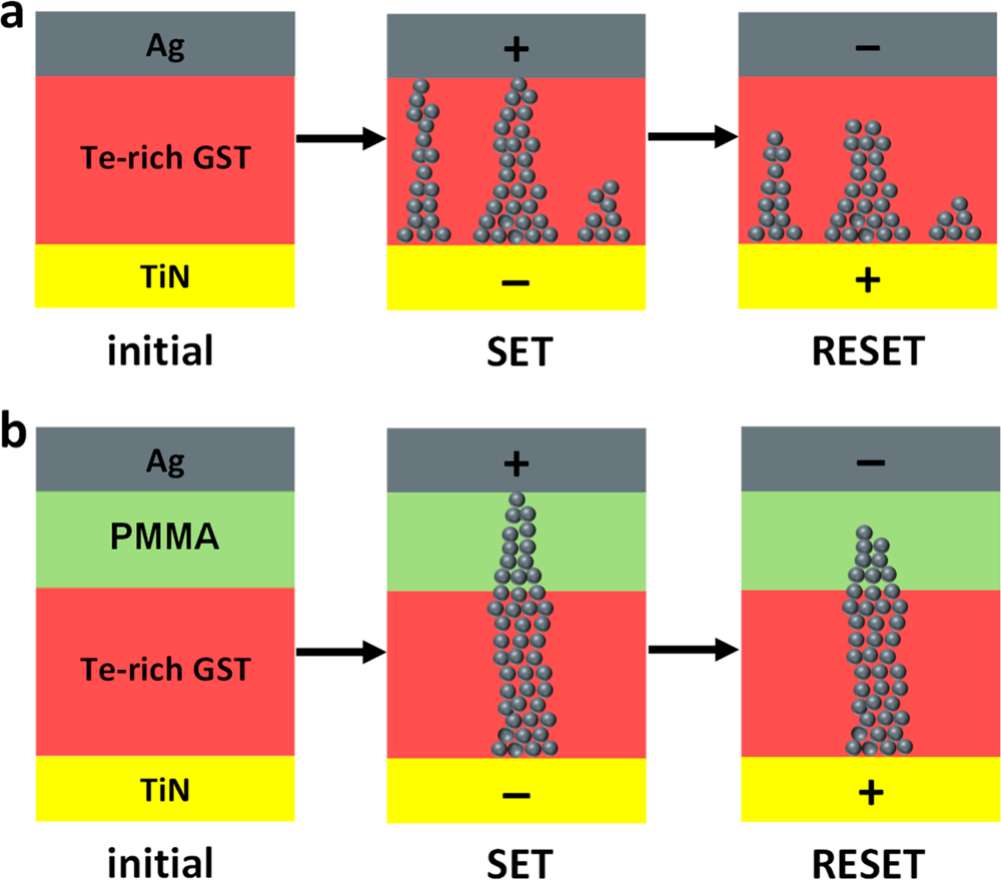
Schematic showing the proposed switching mechanism for (a) the single GeSbTe memristor and (b) the hybrid PMMA/GeSbTe memristor.

A switching mechanism consistent with the electrical data is proposed and displayed in Figure 6. The switching mechanism is based on the formation and rapture of conductive Ag filament(s) across the GeSbTe and the hybrid PMMA/GeSbTe layer upon application of different electric field polarities. In the case of a single-layer GeSbTe-based memristor, the switching mechanism occurs within the GeSbTe thin film, which serves as an electrolyte for the ECM process (Figure 6a).

In the case of the hybrid memristor, both PMMA/GeSbTe matrix can act as electrolyte, where a resistive switching through oxidation/reduction reactions (ECM process) can occur upon altering the polarity of the electric field (Figure 6b). The hybrid device is initially in the HRS (initial). Applying a positive potential to the top Ag electrode ionizes the Ag atoms (Ag^+^) at the top Ag/PMMA interface. Under high electric field, the Ag^+^ ions can move across the PMMA and GeSbTe to the bottom electrode, where the neutral Ag atoms are accumulated and start to form a filament. Once the grown filament connects to the top Ag electrode, the device switches to the LRS (SET). In contrast, applying a negative potential moves the Ag^+^ ions formed at the bottom electrode to the top electrode and ruptures the preformed filament, switching the device back to the HRS (RESET).

The results obtained suggest that the PMMA layer plays a key role in improving the resistive switching properties. As a barrier between the GeSbTe and top electrode, the PMMA diminishes the penetration of the Ag atoms into the voids of the GeSbTe thin film. The PMMA also changes the switching between the two resistance states from a smooth transition for the GeSbTe device to a sharp transition for the hybrid PMMA/GeSbTe devices. For the devices containing only GeSbTe, multiple, large Ag filaments can easily be formed across the high porosity GeSbTe thin film upon application of high electric field. The large Ag filament(s) results in the high operating currents and is also responsible for the CC requirement during the RESET process. For the devices containing hybrid PMMA/GeSbTe material, the formation of Ag filaments across the hybrid material tends to be more restricted at high PMMA concentration, resulting in a sharp switching with 2 orders of magnitude difference between the LRS and HRS state. In addition, the PMMA makes the switching from the HRS to the LRS slower, as the SET voltage increases from ∼0.6 V for the devices containing only GeSbTe thin films to ∼1.7 V for the devices containing hybrid PMMA (50%)/GeSbTe material. The PMMA may also smooth the GeSbTe interface region and mask local surface defects and surface roughness^66^ that can result in undesirable device properties such as switching the device to a higher current level and breakdown in the absence of CC during RESET process.^41^ Protecting the GeSbTe with PMMA not only improves the resistive switching properties but also reduces the standby power consumption, which potentially suppresses the sneak current and devices disturbance issues.^18^

**Figure 7:**
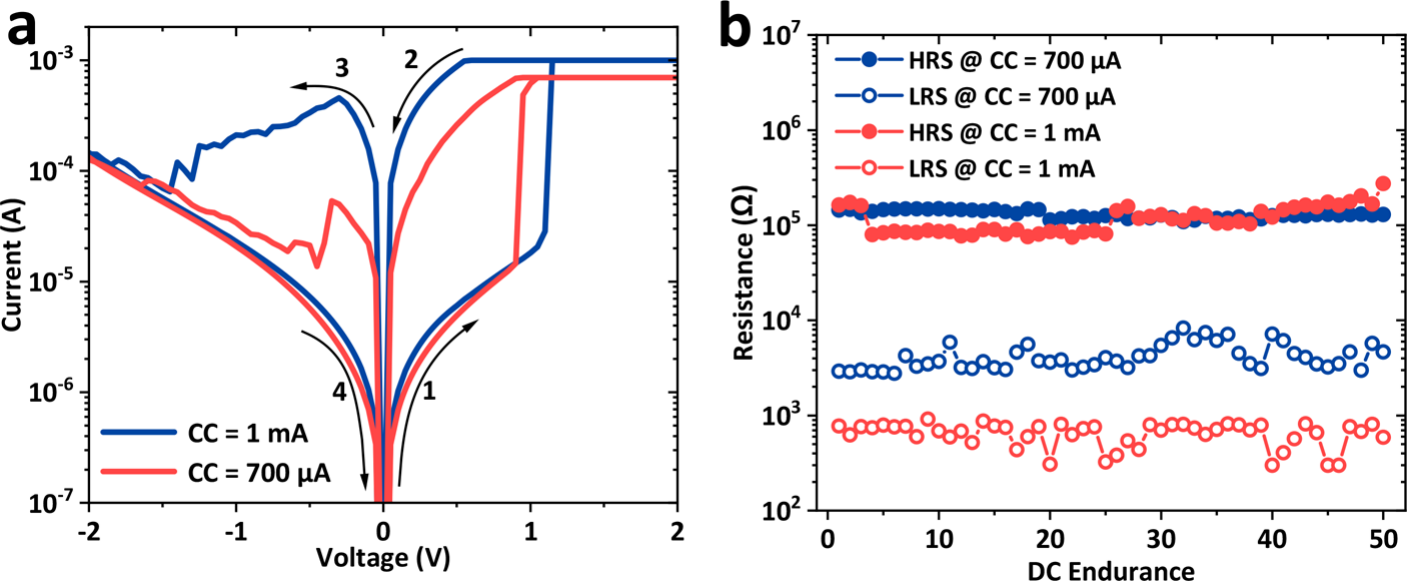
(a) *I*–*V* characteristics of a Ag/PMMA/GeSbTe/TiN memristor (50% PMMA) for different CC. (b) DC endurance of the device showing the reproducible multistate resistance behavior under different CCs.

Multistate switching can be achieved in our hybrid PMMA/GeSbTe devices by controlling the CC level. Figure 7a demonstrates the current–voltage curves of a hybrid Ag/PMMA/GeSbTe/TiN memristor switched at different CC level with a sweep voltage of ±2 V. The plot shows that the device switches to different LRS values upon varying the CC level from 700 μA (red curve) to 1 mA (blue curve), while the HRS remained almost the same. Therefore, the device exhibits three distinct resistance states including one HRS and two LRS. The multistate switching behavior is uniform and reproducible for 50 cycles, as demonstrated in Figure 7b. Such behavior suggests that our hybrid memristor devices have promising potential as candidates of artificial synapses for neuromorphic computing and multilevel data storage applications.^67−70^ Such CC level-dependent control has been widely demonstrated in resistive switching memories and can be ascribed by the formation of filaments with different dimensions across the device.^56,71^ Although similar multistate switching behavior was previously observed in our crossbar GeSbTe-based memory devices, the ON/OFF resistance ratio was smaller (by about 1 order of magnitude), and the CC was needed for both the SET and RESET processes to protect the devices.^41,56,71^

**Figure 8:**
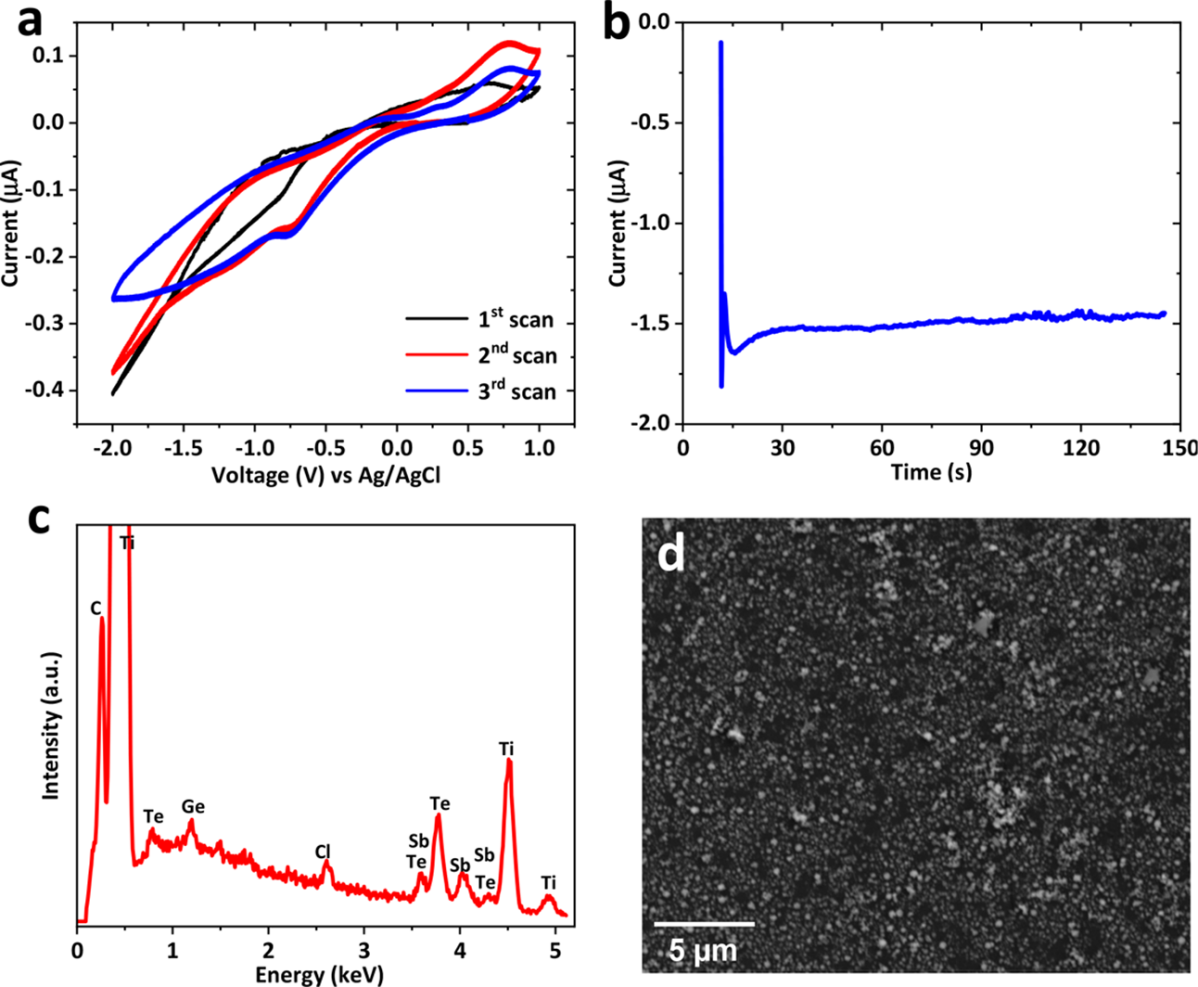
(a) CV of the 4-mm-diameter TiN/polyimide substrate in 0.1 M [N^n^Bu_4_]Cl electrolyte containing 2.5 mM [N^n^Bu_4_][GeCl_5_], 1 mM [N^n^Bu_4_][SbCl_4_], and 2 mM [N^n^Bu_4_]_2_[TeCl_6_]. Scan rate: 50 mV s^–1^. (b) Current time transient for GeSbTe electrodeposition on polyimide substrate at deposition potential of −1.75 V vs Ag/AgCl. The cutoff charge is −5 μC. (c) EDX spectrum of the electrodeposited GeSbTe on polyimide substrate showing the existence of Ge, Sb, and Te within the thin film. (d) SEM image of the GeSbTe thin film on TiN/polyimide substrate.

We will now demonstrate the development of hybrid PMMA/GeSbTe material-based memristor devices on flexible substrate. The GeSbTe thin film was electrodeposited on a polyimide substrate with a TiN layer as the bottom electrode. The cyclic voltammetry and deposition transient are shown in Figure 8a and b, respectively. The anodic stripping region of the cyclic voltammetry is similar to the deposition on Si supported TiN substrates in Figure 2; however, the peaks at the cathodic deposition region are less obvious on the flexible TiN substrates for the first scan. Subsequent CVs are similar to those shown in Figure 2a. The actual deposition is controlled by the same cutoff charge of −5 μC to achieve a nominal thickness of 100 nm. The GeSbTe composition of 7:20:73 was identified by EDX (shown in Figure 8c). The EDX spectrum also shows the presence of Cl, which is originated from the electrolyte. Top SEM image of the electrodeposited GeSbTe thin film is shown in Figure 8d. Furthermore, AFM image of the film is presented in Figure S4. The 33% PMMA concentration solution was selected to deposit the PMMA layer on the GeSbTe thin film to obtain the hybrid material structure.

**Figure 9:**
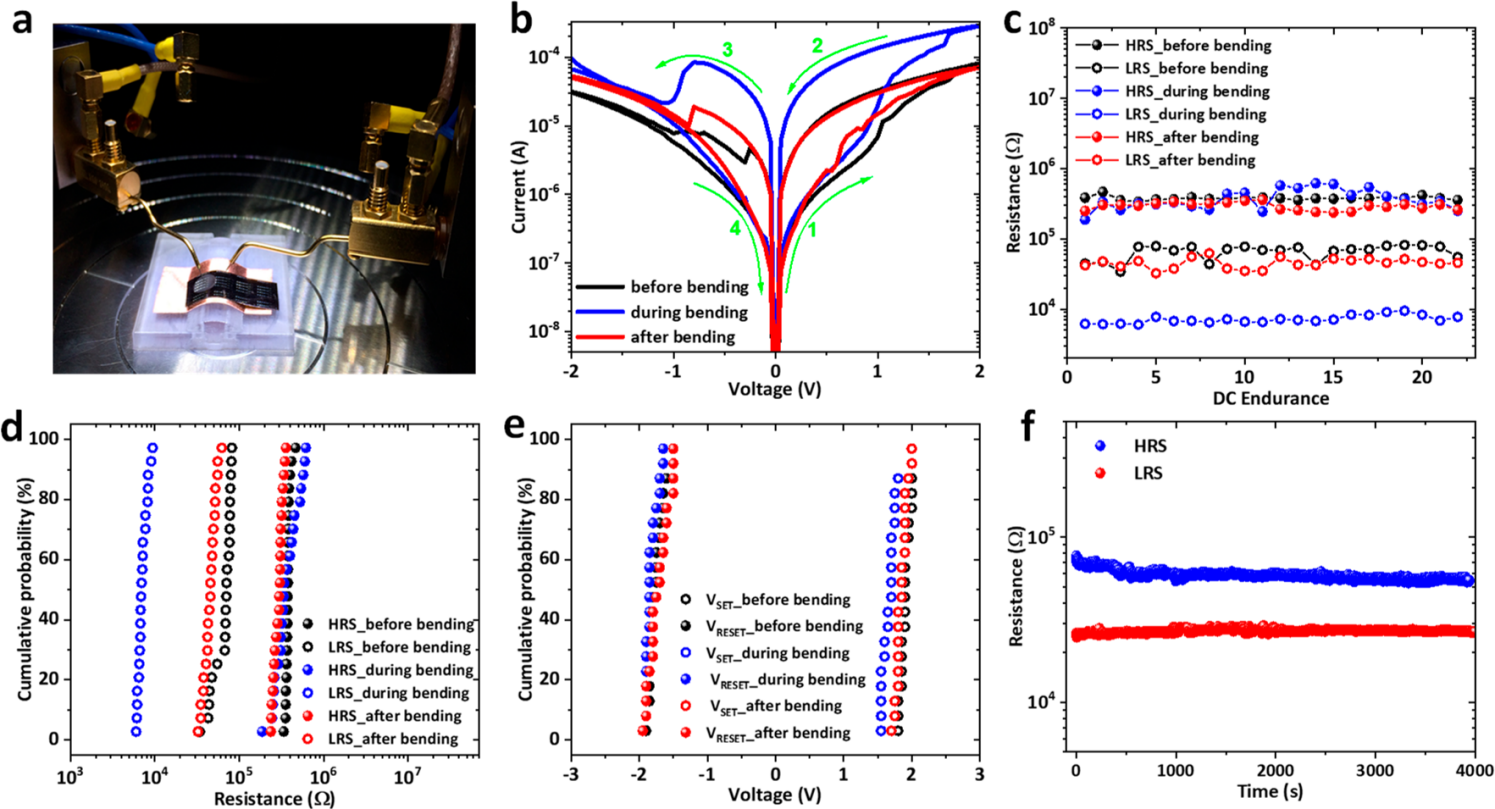
(a) A real photo image of a hybrid flexible memristor under bending radius (*R*) of 15 mm. (b) *I*–*V* resistive switching characteristics of a flexible Ag/PMMA/GeSbTe/TiN device before bending (black curve), during bending (blue curve), and after bending (red curve). (c) Endurance characteristics of the device at a read voltage of 0.5 V. (d) Cumulative probability of HRS and LRS, and (e) *V*_SET_ and *V*_RESET_. (f) Retention test for the device after bending at a read voltage of 0.5 V.

A photographic image of the developed flexible hybrid memory devices with the structure of Ag/PMMA/GeSbTe/TiN under bending is shown in Figure 9a. In order to confirm the feasibility of our devices for flexible nonvolatile memory applications, mechanical flexibility test was carried out on a curved surface with a bending radius (*R*) of 15 mm. The effect of mechanical flexibility testing on the electronic properties of the flexible memristor device was investigated by performing *I*–*V* sweeps before bending (black curve), during bending (blue curve), and after bending (red curve), as shown in Figure 9b. The device shows a bipolar resistive switching behavior with the SET and RESET processes occurred at positive and negative voltage polarities, respectively. The device exhibited reliable operation even under bending condition. It is interesting to note that the ON current is shifted to a higher level, increasing the ON/OFF ratio from 10 under flat condition to 33 under bending condition. The effect is reversible, and the ON current shifts back to its original starting position after bending. The higher ON current obtained here is likely to be induced by stretching the PMMA material upon bending, which could reduce the thickness of the PMMA and as a result reduces the resistance of the device.

To evaluate operational reliability of our hybrid flexible memristor, the memory performance properties including the DC endurance and cumulative probability for the ON/OFF resistance ratio were analyzed. As shown in Figure 9c, the device showed a stable endurance in the DC mode for both the HRS and LRS, and no degradation was observed under either flat or bending conditions. Figure 9d shows the high uniformity of cumulative probability distributions for the HRS and LRS under flat and bending conditions. Moreover, to examine the operational switching voltages uniformity of the device, cumulative probability distributions for the *V*_SET_ and *V*_RESET_ were also analyzed under flat and bending conditions as shown in Figure 9e. The plot shows excellent uniformity distribution for both switching voltages, which are desirable for nonvolatile RRAM applications. To further evaluate the reliability and stability of the device after bending conditions, retention performance was examined for the HRS and LRS at a continuous voltage of 0.5 V. Both HRS and LRS were found to be retained without degradation for over 4000 s, indicating the nonvolatility and good retention characteristics of the device as shown in Figure 9f. Further DC sweeps from another device also show a reproducible switching performance before, during, and after bending are reported in Supporting Information Figure S5. This suggests electrodeposition is a viable approach for fabricating flexible memristor devices.

## 3 Conclusions

In conclusion, we have successfully demonstrated the development of a flexible memristor that shows reliable bipolar resistive switching properties at low switching voltages. This is achieved by utilizing a hybrid organic–inorganic materials approach by incorporating PMMA with resistive switching electrodeposited GeSbTe nanoscale thin films. The switching mechanism in these devices is explained by the formation and rapture of conductive Ag filament(s) upon application of different electric field polarity. The resistance states can be controlled by CC, demonstrating the multistate switching behavior. Our approach for the fabrication of flexible memristor devices could have potential applications in future nonvolatile data storage electronics.

## 4 Experimental Section

The memristor devices consist of a vertical thin film stack with the following order of materials, Ag/PMMA/GeSbTe/TiN (Figure 3a). Prior to electrodeposition process of GeSbTe thin films, TiN bottom contacts (200 nm thick) were sputtered on silicon and polyamide substrates. The GeSbTe thin films were deposited on these substrates using the following procedure.

### Solution

Electrolytes were prepared in anhydrous CH_2_Cl_2_ (Sigma-Aldrich, 95%), dried and degassed by refluxing with CaH_2_, followed by distillation and then stored in the glovebox. A 0.1 M [N^n^Bu_4_]Cl (Sigma-Aldrich, ≥ 99.0%, dried) was used as the supporting electrolyte. The electrolyte contains 2.5 mM [N^n^Bu_4_][GeCl_5_], 1 mM [N^n^Bu_4_][SbCl_4_], and 2 mM [N^n^Bu_4_]_2_[TeCl_6_], which were synthesized as described previously.^61^

### Electrodes

Silicon and polyamide substrates with predeposited TiN bottom electrodes serve as the electrode for electrodeposition. The detailed fabrication process can be found in our previous work.^47,72^ A Pt/Ir (90%:10%) disk was employed as the counter electrode and an Ag/AgCl wire (0.1 M [N^n^Bu_4_]Cl in anhydrous CH_2_Cl_2_) was used as the reference electrode.

### Electrochemistry

All cyclic voltammetry (CV) and electrodeposition experiments were carried out in a recirculating glovebox (Belle) to exclude moisture and air. Oxygen levels were kept below 10 ppm. A microAutolab 3 potentiostat and a Nova 1 software package were used for all electrochemical measurements. Prior to GeSbTe electrodeposition, voltammetry was recorded on a TiN bottom contact in CH_2_Cl_2_ solution containing 2.5 mM [N^n^Bu_4_]GeCl_5_, 1 mM [N^n^Bu_4_]SbCl_4_, and 2 mM [N^n^Bu_4_]_2_TeCl_6_ at a scan rate of 50 mV s^–1^, Figure 2a. On the first scan reduction starts at around −1 V, and there is evidence of a nucleation loop, consistent with deposition on the electrode surface, on the return scan. Similar behavior is seen on the second and third scans, although now the reduction current increases less steeply, possibly due to the presence of a semiconducting deposit of GeSbTe at the electrode surface. On the return scan a stripping peak is evident at +0.5 V. This voltammetry is broadly consistent with our earlier work.^47^ Electrodeposition of GeSbTe was carried out at −1.75 V vs Ag/AgCl with a cutoff charge of 5 μC to allow a complete depositing GeSbTe thin film, as shown in Figure 2b. Electrodeposition was carried out by holding the TiN electrode at open circuit potential for the initial 5 s, followed by applying −1.75 V vs Ag/AgCl. The measurement stops automatically when the charge passed at the working electrode reaches 5 μC. The substrate was then rinsed in CH_2_Cl_2_ and stored in the glovebox.

### Device Fabrication and Characterization

495 poly(methyl methacrylate) (PMMA) solution, purchased from Sigma-Aldrich, was diluted in anisole in different ratios designed to give predefined PMMA concentration solutions, 20%, 25%, 33%, and 50%. The solutions were spin coated onto the pre-electrodeposited GeSbTe thin films at 4000 rpm for 60 s. The samples were then annealed at 120 °C for 3 min in air to remove any remaining solvent. Ag top electrodes (200 nm thick) were thermally deposited under vacuum conditions through a shadow mask containing 250-μm-diameter circles. Note that a control device consisting of GeSbTe (no PMMA) with a structure of Ag/GeSbTe/TiN was also fabricated. The electrical characteristics were measured at room temperature and ambient pressure using a probe connected to a Keysight (B1500) system. For all measurements, the voltage was applied to the top electrode Ag contacts, while the bottom electrode TiN contacts were grounded. SEM, EDX and AFM were used to characterize the thin films.

## Supporting Information Available

Extra details of thickness characterization, electrical characterization, and AFM image (PDF)

## Notes

Data supporting this study are openly available from the University of Southampton repository at DOI: https://doi.org/10.5258/SOTON/D2440. Additional data from this study are available from the corresponding author upon reasonable request.
