# Supplementary material for: Flexible Memristor Devices Using Hybrid Polymer/Electrodeposited GeSbTe Nanoscale Thin Films
Source: ACS Appl Nano Mater. 2022 Nov 25;5(12):17711–20. doi: 10.1021/acsanm.2c03639 (PMC9791617; doi:10.1021/acsanm.2c03639)
Supplement: Supplementary file 1 — an2c03639_si_001.pdf [file an2c03639_si_001.pdf]

Supporting Information

Flexible Memristor Devices Using Hybrid Polymer/Electrodeposited GeSbTe Nanoscale Thin Films

Ayoub H. Jaafar,[a,b,] Lingcong Meng,[c,d] Tongjun Zhang,[a] Dongkai Guo,[a] Daniel Newbrook,[a] Wenjian Zhang,[c] Gillian Reid,[c] C.H. (Kees) de Groot,[a] Philip N. Bartlett,[c,*] and Ruomeng Huang[a,*]*

[a] School of Electronics and Computer Science, University of Southampton, Southampton, SO17 1BJ, UK

[b] School of Physics and Astronomy, University of Nottingham, Nottingham, NG7 2RD, UK

[c] School of Chemistry, University of Southampton, Southampton, SO17 1BJ, UK

[d] School of Chemistry, University of Lincoln, Lincoln, LN6 7TS, UK

*a.h.j.hamdiyah@soton.ac.uk; p.n.bartlett@soton.ac.uk; r.huang@soton.ac.uk

The relationship between the PMMA polymer film thickness and concentration of solutions was studied, Figure S1. The PMMA concentration solutions, 20%, 25%, 33%, and 50%, were spin coated onto Si substrates at 4000 rpm for 60 s. The samples were then annealed at 120 ˚C for 3 min in air to remove any remaining solvent. The thickness of PMMA films were measured by using a Dektak (Bruker) surface profilometer. From the graph, it can be seen that increasing the solution concentration increases the film thickness from 15 nm to 160 nm. We expect the thickness of PMMA deposited on GeSbTe thin films is thinner due to differences in the wettability and frictional forces of the GeSbTe surface in contrast to Si.

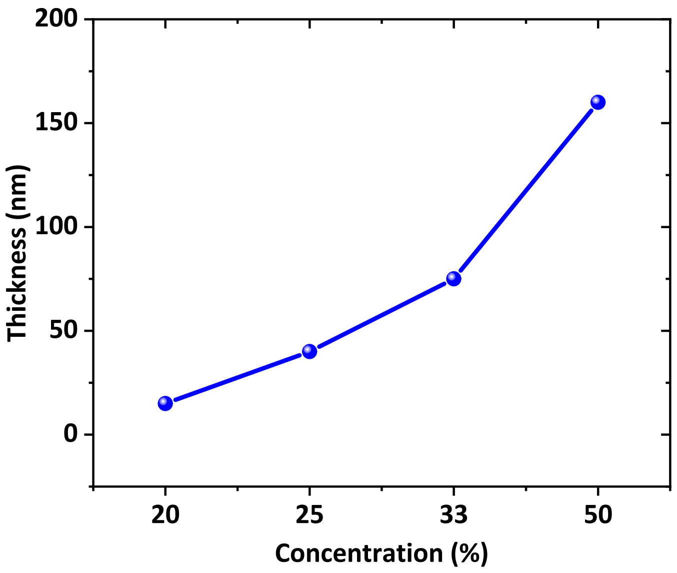

Figure S1. The thickness of PMMA layer as a function of solution concentration. The solutions were spin coated on Si substrates at 4000 rpm for 60 sec and then annealed at 120 ˚C for 3 min in air to remove any remaining solvent.

To examine stability of the ON and OFF states, the DC endurance tests were carried out on devices with different PMMA concentration solutions as shown in Figure S2a-e. Further, data retention test, Figure S2f, for both ON and OFF states was examined for a device with 50% PMMA concentration after setting the device to LRS (black data) and to HRS (red data). The endurance and retention tests show that both ON and OFF states are stable and maintained over the length of the experiment without considerable resistance degradation.

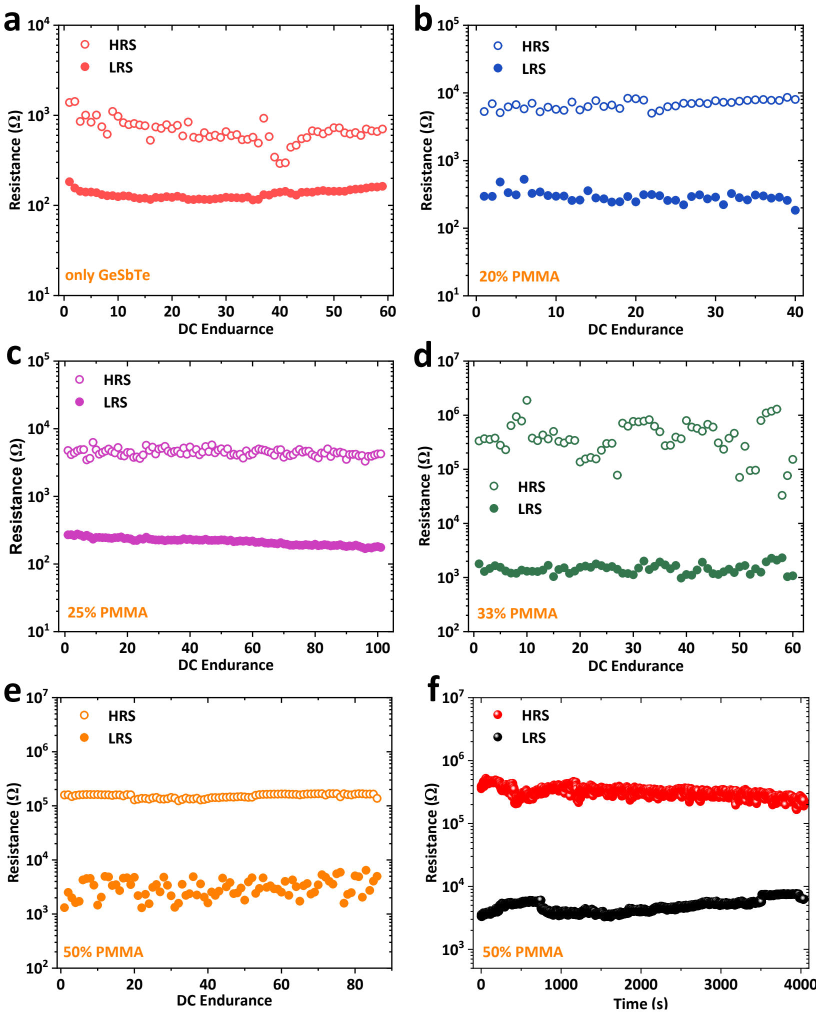

Figure S2. (a-e) DC endurance characteristics for HRS and LRS at a read voltage of 0.1 V. (f) Retention time test for 4000 s for the HRS and LRS at a read voltage of 0.1 V for a hybrid GeSbTe/PMMA memristor device.

Great clarity of the current transport mechanism can be observed from re-plotting the data on a log-log *I-V* plot in Figure S3. Different regions of conduction with different slopes can be identified upon transition from the high resistance state (HRS) to the low resistance state (LRS), showing a space charge limited current (SCLC) mechanism.

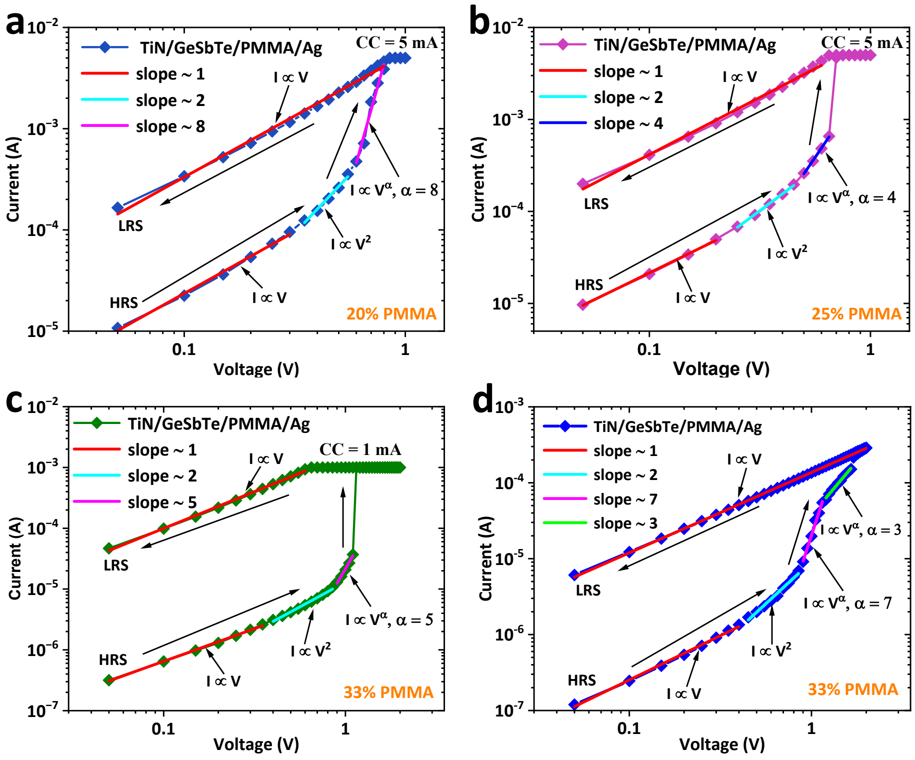

Figure S3. Current−Voltage curves demonstrating fits to the space charge limited current (SCLC) mechanism for the HRS to LRS transition for hybrid TiN/PMMA/GeSbTe/Ag

memristor devices fabricated on Si substrates (a-c) at different concentrations of PMMA, and on polyimide substrate (d) at 33% PMMA concentration.

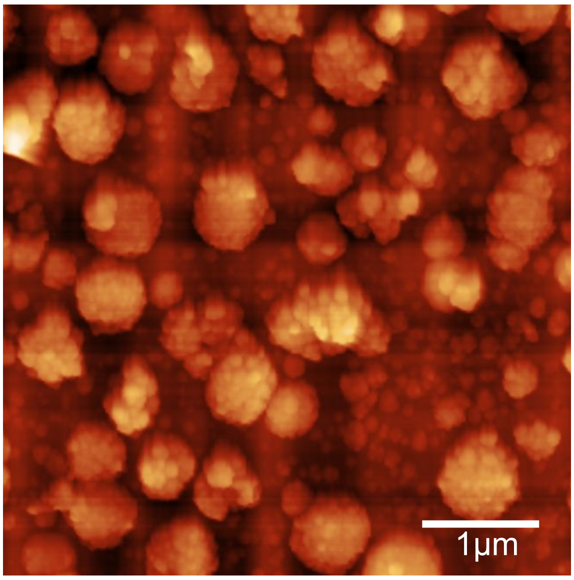

Figure S4. AFM image for the electrodeposited GeSbTe thin film on polyimide substrate.

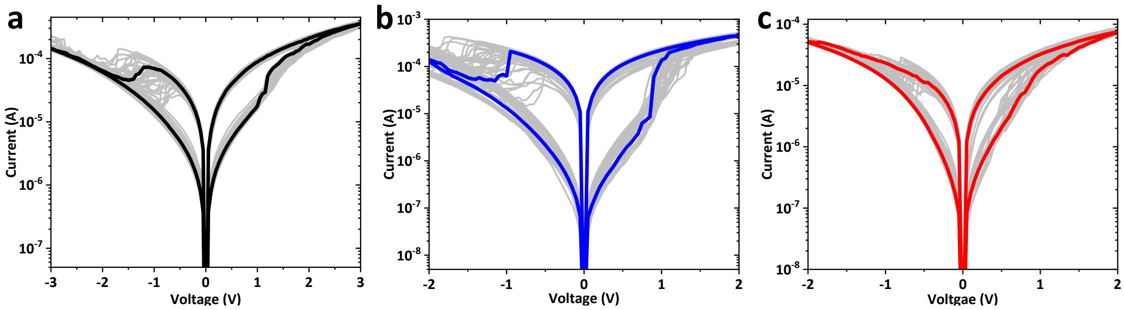

Figure S5. Consecutive current-voltage (I-V) characteristics of flexible hybrid memristor devices, TiN/PMMA/GeSbTe/Ag, taken before bending (a) during bending with a bending radius of 15 mm (b) and after bending (c). The I-V characteristics show a reproducible switching performance.
